# Assessing the immunogenicity risk of salmon calcitonin peptide impurities using *in silico* and *in vitro* methods

**DOI:** 10.3389/fphar.2024.1363139

**Published:** 2024-08-09

**Authors:** Brian J. Roberts, Aimee E. Mattei, Kristina E. Howard, James L. Weaver, Hao Liu, Sandra Lelias, William D. Martin, Daniela Verthelyi, Eric Pang, Katie J. Edwards, Anne S. De Groot

**Affiliations:** ^1^ EpiVax Inc., Providence, RI, United States; ^2^ Division of Applied Regulatory Sciences, Office of Clinical Pharmacology, Office of Translational Sciences, Center for Drug Evaluation and Research, U.S. Food and Drug Administration, Silver Spring, MD, United States; ^3^ Division of Therapeutic Performance I, Office of Research and Standards, Office of Generic Drugs, Center for Drug Evaluation and Research, U.S. Food and Drug Administration, Silver Spring, MD, United States; ^4^ Division of Biotechnology Review and Research III, Office of Biotechnology Products, Office of Pharmaceutical Quality, Center for Drug Evaluation and Research, U.S. Food and Drug Administration, Silver Spring, MD, United States; ^5^ CUBRC, Inc., Buffalo, NY, United States

**Keywords:** salmon calcitonin, peptide drug, impurity, immunogenicity, computational immunology, T-cell epitope, HLA binding, T-cell assay

## Abstract

Advances in synthetic peptide synthesis have enabled rapid and cost-effective peptide drug manufacturing. For this reason, peptide drugs that were first produced using recombinant DNA (rDNA) technology are now being produced using solid- and liquid-phase peptide synthesis. While peptide synthesis has some advantages over rDNA expression methods, new peptide-related impurities that differ from the active pharmaceutical ingredient (API) may be generated during synthesis. These impurity byproducts of the original peptide sequence feature amino acid insertions, deletions, and side-chain modifications that may alter the immunogenicity risk profile of the drug product. Impurities resulting from synthesis have become the special focus of regulatory review and approval for human use, as outlined in the FDA’s Center for Drug Evaluation and Research guidance document, “ANDAs for Certain Highly Purified Synthetic Peptide Drug Products That Refer to Listed Drugs of rDNA Origin,” published in 2021. This case study illustrates how *in silico* and *in vitro* methods can be applied to assess the immunogenicity risk of impurities that may be present in synthetic generic versions of the salmon calcitonin (SCT) drug product. Sponsors of generic drug abbreviated new drug applications (ANDAs) should consider careful control of these impurities (for example, keeping the concentration of the immunogenic impurities below the cut-off recommended by FDA regulators). Twenty example SCT impurities were analyzed using *in silico* tools and assessed as having slightly more or less immunogenic risk potential relative to the SCT API peptide. Class II human leukocyte antigen (HLA)-binding assays provided independent confirmation that a 9-mer sequence present in the C-terminus of SCT binds promiscuously to multiple HLA DR alleles, while T-cell assays confirmed the expected T-cell responses to SCT and selected impurities. *In silico* analysis combined with *in vitro* assays that directly compare the API to each individual impurity peptide may be a useful approach for assessing the potential immunogenic risk posed by peptide impurities that are present in generic drug products.

## Introduction

The US generic drug market was valued at 62 billion USD in 2023 and expected to increase in the future, as more generics are approved (IQVIA Institute of Human Data Science, 2027). Due to advances in synthetic peptide synthesis that allow for more rapid and cost-effective manufacturing, combined with improved analytical techniques, peptide drugs that were once manufactured by recombinant DNA technology can now be produced using solid- and liquid-phase synthesis. However, a byproduct of peptide synthesis is the generation of new peptide-related impurities. Additionally, degradation product impurities may result from factors such as formulation, storage conditions, and container closure, leading to impurities independent of the manufacturing process. The resulting impurities may have an impact on the safety and efficacy of the final drug product. In addition, contaminants, such as extractables and leachables, may also be present in the final drug formulation and may have an impact on new product degradants. This paper discusses peptide-related impurities that could be presented in the context of the human leukocyte antigen (HLA) or alter the binding of the active pharmaceutical ingredient (API) to the histocompatibility complex (MHC) and lead to a *de novo* T-cell response that could impact the immunogenicity (and safety) of the final product.

Peptide-related impurities include modifications to the API peptide that are introduced during the synthesis process due to 1) failures in peptide synthesis: amino acid insertions and duplications, amino acid deletions, and racemization; 2) contaminated raw materials leading to the incorporation of unnatural amino acids and insertion of β-amino acids; and 3) incomplete removal of protecting groups that can result in unintentional side-chain modifications. Additionally, peptide-related impurities can result from post-synthesis degradation, leading to aggregation and side-chain modifications such as oxidation and deamidation of susceptible amino acid residues. Peptide-related impurity modifications can also result in the introduction of new T-cell epitopes, not present in the API, which may induce an unwanted immune response to the impurity, causing an anti-drug immune response to occur and impacting both the safety and efficacy of the drug product.

The presence of impurities in synthetically prepared generic peptide products referencing recombinant glucagon, liraglutide, nesiritide, teriparatide, and teduglutide is addressed in the United States Food and Drug Administration (FDA) document, “*ANDAs for Certain Highly Purified Synthetic Drug Products That Refer to Listed Drugs of rDNA Origin*” (Center for Biologics Evaluation and Research CDER, 2021). Abbreviated new drug application (ANDA) refers to an approval pathway for drugs that eliminates the need to repeat clinical trials for a generic drug that is shown to be the same as an approved drug. This guidance recommends that generic drug applicants aiming to obtain ANDA approval for their generic peptides identify and describe all peptide impurities present at a concentration greater than 0.10% of the API in the final drug product and determine if these impurities are different from those found in the reference listed drug (RLD). Furthermore, ANDA applicants are recommended to assess whether any peptide impurities that are different from those in the RLD, or present at a higher concentration than found in the RLD, could increase the risk of immunogenicity of the proposed generic drug compared to that of the RLD.

Although the immunogenicity of new peptide drug products is typically assessed clinically by measuring anti-drug antibodies (ADAs) in the patient population, the immunogenicity risk potential or immunogenicity risk profile of generic peptide drugs can be informed by existing information about the RLD. Hence, under the ANDA filing pathway, a thorough characterization of the product and its impurities, including any novel HLA ligands (and potential T-cell epitopes) that may be present within the sequence of the peptide-related impurities, is recommended. Most generic peptide drug impurities can be evaluated for immunogenicity risk using *in silico* tools, and their immunogenic risk potential can also be independently assessed in parallel in *in vitro* studies (Jawa et al., 2020). A detailed description of methods that apply to all peptides is provided by De Groot et al. (2023).

*In vivo,* peptide drugs undergo endocytosis by antigen-presenting cells (APCs), such as dendritic cells (DCs), and are cleaved into smaller peptide fragments that are presented to helper T cells on class II HLA molecules expressed on the surface of the APC. Upon recognition of the peptide–HLA complex by the T-cell receptor, the T cells will become activated and provide the necessary stimulus for B cells to mature and produce antibodies. This is the mechanism by which peptide impurities containing new T-cell epitopes may drive unwanted immune responses. In the absence of T-helper epitopes, helper T cells fail to activate antigen-specific B-cell maturation, reducing antibody class switching and the formation of ADAs (Duke and Mitra-Kaushik, 2019; Jawa et al., 2020).

**FIGURE 1:**
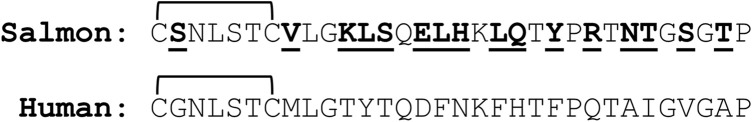
Comparison of the salmon (SCT) and human calcitonin sequences. Amino acids in SCT that are bold and underlined differ between the two forms of the peptide. The bracket connecting cysteines 1 and 7 indicates the disulfide bond that forms a ring structure in the N-terminus of the peptide (Kozono et al., 1992).

In this case study, we describe an exercise to assess the immunogenicity risk of generic peptide impurities that were identified in salmon calcitonin (SCT). SCT is a peptide drug currently under development for the generic market by several manufacturers. Salmon calcitonin is a 32-amino acid peptide drug approved in the United States for the treatment of postmenopausal osteoporosis, Paget’s disease, and hypercalcemia. Salmon calcitonin shares only 50% amino acid sequence homology with human calcitonin (Figure 1), and despite its therapeutic benefits, in clinical trials, 40%–70% of patients treated with SCT develop ADAs within 4 months of treatment. While not all of these patients develop neutralizing anti-SCT antibodies (NADAs), more than 60% of those who develop ADAs do, causing them to become resistant to SCT therapy, ultimately requiring alternative forms of treatment (LEVY et al., 1988; Grauer et al., 1995).

Despite the risk of immunogenicity, SCT is preferred for therapeutic use over human calcitonin due to its 50-fold greater potency *in vivo*. Increased potency is attributed to the ability of the peptide to adopt an α-helical structure and bind to the human calcitonin receptor with a greater, and nearly irreversible, affinity relative to the human homolog (Epand et al., 1983; Lee et al., 2011; Andreassen et al., 2014).

The API in the salmon calcitonin RLD, Miacalcin^®^, is produced using recombinant DNA (rDNA) technology, while most generic SCT products are produced by synthetic peptide synthesis. Due to the differences in the manufacturing processes, the impurity profile of synthetically produced peptide drugs could differ from those in the RLD; thus, an assessment of the impurities that are present in the synthetic drug product is usually required for approval of the generic drug product.

**FIGURE 2:**
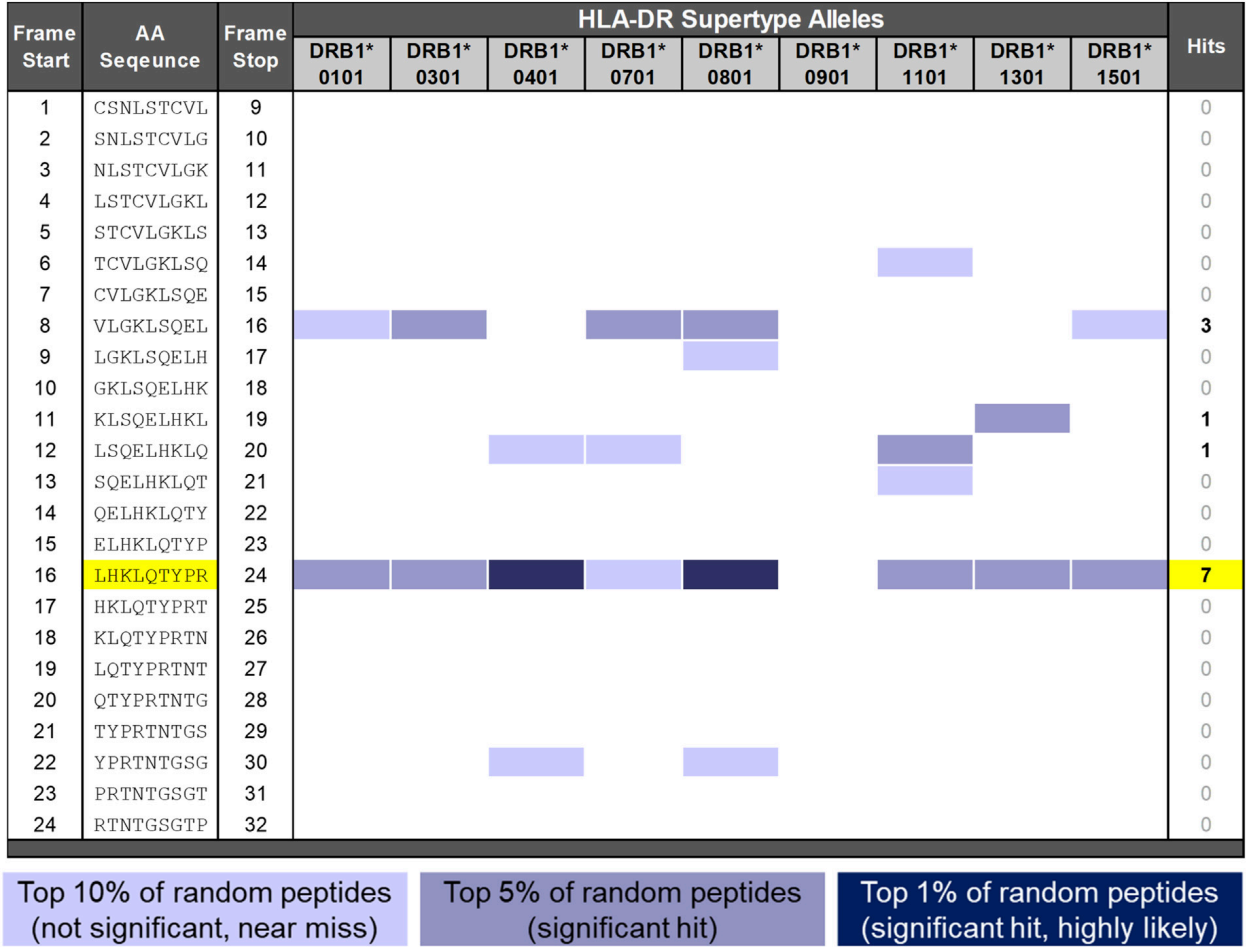
EpiMatrix analysis of SCT. The potential of a 9-mer frame to bind to a given human leukocyte antigen (HLA) allele is indicated by a Z-score (scores omitted for simplicity); the strength of the score is indicated by the blue shading. All scores in the top 5% of the normal distribution are considered “hits” (medium- and dark-blue shading). Scores in the top 10% are considered elevated but not significant (light blue shading). Frames containing four or more alleles scoring in the top 5% and above are referred to as EpiBars and are highlighted in yellow. These frames have an increased likelihood of binding to a range of HLA alleles. The 9-mer epitope bar highlighted in yellow is a putative promiscuous epitope.

While salmon calcitonin is known to induce ADA production *in vivo*, less is known about the T-cell response to the peptide. In a previous study, Tangri et al. (2005) identified a region located within the center of SCT, GKLSQELHKLQTYPRT, that contains a T-cell epitope shown to bind to HLA DRB1*0101 and *0401. It is important to note that this epitope resides within a region of SCT that differs from human calcitonin by 10 of the 16 amino acids. This 9-mer frame (frame 16 in Figure 2) is contained near regions previously identified as T- and B-cell epitopes by Tangri and Kozono, respectively (Kozono et al., 1992; Tangri et al., 2005), although the two types of epitopes do not overlap with the N-terminal region.

To assess the utility of *in silico* and *in vitro* immunogenicity risk screening tools for the evaluation of peptide drug impurities, the FDA Center for Drug Evaluation and Research (CDER) provided EpiVax with a list of 20 peptide-related salmon calcitonin impurities identified as byproducts of synthetic salmon calcitonin synthesis or degradation under FDA contract HHSF223018186C. The immunogenicity risk was assessed utilizing three independent (orthogonal) methods: *in silico* analysis, class II HLA binding assays, and naïve T-cell assays (De Groot et al., 2023). The *in silico* analysis indicated that immune responses to SCT are likely to be related to the presence of a 9-mer binding frame, which is not conserved with any human protein and contains a promiscuous HLA DR-binding T-cell epitope. This 9-mer frame overlaps with the epitopes previously identified by Kozono et al. (1992) and Tangri et al. (2005). The selected SCT impurities were compared to the API, and an *in silico* assessment was performed to identify new (putative) T-cell epitopes in the impurity sequences.

*In vitro* HLA-binding and T-cell assays were used to determine whether any of the 20 impurities had the potential to be immunogenic. One key finding reported here is that, consistent with the high incidence of ADAs in the patients treated with SCT, the API peptide elicited naïve T-cell responses from multiple donors, *in vitro*, on its own. Also consistent with the *in silico* analysis, the number of donor peripheral blood mononuclear cell (PBMC) samples responding to the peptide impurities was increased, when compared to donor PBMC sample responses to the SCT API peptide, in the naïve CD4 T-cell assays.

Taken together, the results indicate that the *in vitro* class II HLA binding and T-cell assays that were performed using the API peptide and selected impurities were generally aligned with the *in silico* risk assessments of the same sequences, suggesting that this approach of combining *in silico* and *in vitro* evaluation methods is useful for evaluating the immunogenicity risk of peptide impurities, as recommended in the FDA guidance document, “*ANDAs for Certain Highly Purified Synthetic Drug Products That Refer to Listed Drugs of rDNA Origin*.”

## Material and methods

### Bioinformatics analysis screening of salmon calcitonin and impurities

A wide range of tools have been developed and applied to therapeutic proteins and peptides for identifying T-cell epitopes. Here, we describe the application of tools developed by EpiVax; however, alternative *in silico* tools can be used to achieve similar results (Supplementary Table S1). First, the SCT API and impurities were evaluated for immunogenic potential using the EpiMatrix T-cell epitope-mapping and JanusMatrix human homology algorithms (De Groot et al., 2003; Moise et al., 2013). In some instances, impurity sequences contained unnatural or otherwise modified amino acid residues. The HLA-binding properties of peptides containing chemically modified or unnatural amino acids could not be directly estimated by the EpiMatrix system. In those instances, a three-step approach was applied to identify potential natural amino acid substitutions, enabling existing *in silico* tools to assess potential HLA binding. The three-step approach to the *in silico* analysis of sequences containing unnatural amino acids is described in detail in a recent publication (Mattei et al., 2022).

Using EpiMatrix to search for T-cell epitopes, the SCT API and 20 impurity sequences were parsed into overlapping 9-mer frames, where each frame was evaluated for potential binding to a panel of 9 HLA DR supertype alleles (HLA-DRB1*0101, *0301, *0401, *0701, *0801, *0901, *1101, *1301, and *1501) for binding likelihood. Taken together, these nine supertype alleles, along with their respective family members, cover greater than 95% of HLA types present in most human population groups (Southwood et al., 1998; Mattei et al., 2024). The method used to identify and calculate the HLA DR coverage described above is described in detail in Drug Discovery Today by De Groot et al. (2023). HLA DR is selected for the *in silico* analysis of adaptive T-cell response as it has consistently been the most prevalent HLA allele associated with the immunogenicity of biologic products (Hyun et al., 2021; Ramarathinam and Purcell, 2021). Summing across the collective 9-mer frames by HLA DR allele assessments, an EpiMatrix Peptide Immunogenicity Score can be obtained. This score is a measure of the predicted T-cell epitope content contained within the peptide (Moise et al., 2015). While the overall score provides a measurement of the global immunogenic potential, it is also important to assess the peptides for regional immunogenic potentials, specifically for the presence of promiscuous T-cell epitopes. Regional analysis using the HLA DR supertypes in EpiMatrix sometimes reveals an Epitope Bar (EpiBar) feature, which refers to a single 9-mer frame likely to bind at least four different HLA DR supertype alleles. In general, it is expected that these promiscuous class II HLA ligands are the most likely regions of a given peptide or protein to induce CD4^+^ T-cell response.

Next, to identify specific homologies with human proteome T-cell epitopes that may reduce immunogenic potentials, an algorithm called JanusMatrix was used to analyze the impurity sequence epitopes. For any of the putative T-cell epitopes identified in the API and its impurities, the JanusMatrix algorithm identified cross-conserved T-cell epitopes with the same HLA restriction, which are present in the human proteome (thus immunologically related to but not necessarily identical to the input peptide). T-cell epitopes, which are extensively conserved with other peptides in common human proteins, as defined using JanusMatrix, are more likely to be tolerated and may even be tolerogenic in healthy human subjects (Moise et al., 2013; De Groot et al., 2021; 2023). The immunoinformatics tools used in the evaluation of generic drug peptides and their impurities are discussed by De Groot et al. (2023).

Once the *in silico* identification of putative HLA ligands and their conservation within the human proteome were assessed, the next step of the analysis is to compare the API sequence to the impurities to quantify the number of putative new T-cell epitopes present in the impurity sequences, but not the API sequence. In some cases, the modified amino acid sequences of product impurities may include amino acid patterns that are capable of binding to HLA molecules where the unmodified amino acid sequence may not bind. In other cases, modifications may alter the TCR-facing contours of HLA ligands already present in the amino acid sequence of the unmodified product. These modifications often affect serial frames in the sequence. In these instances, all of the newly created epitopes, whether sequential HLA-binding epitopes or sequential T-cell receptor (TCR)-facing epitopes, are considered to be “new epitopes” that can contribute to the potential immunogenicity of the impurity. Modification of the epitope sequence can also “twist” the peptide configuration in the MHC-binding groove, leading to changes in the TCR-facing residues that may impact immunogenicity as well (Singh et al., 2020).

### Selecting salmon calcitonin and impurity peptides for *in vitro* assays

The 20 impurity peptides used in these studies were selected from a survey of SCT impurities that were identified in commercially available nasal salmon calcitonin products and provided by the Office of Generic Drugs (OGD, within the CDER, at the FDA). A detailed list of the 20 impurities is provided in Supplementary Table S2. Of the 20 impurities evaluated *in silico*, a smaller subset was selected for evaluation in independent *in vitro* assays. Each of the impurities contained a single modification relative to the salmon calcitonin baseline sequence.

#### Peptides for HLA-binding assays

SCT impurities that featured modifications to putative HLA-binding residues were selected for *in vitro* HLA DRB1-binding assays. This method was used to assess the impact of the modification on the HLA binding affinity, relative to the baseline. Since most class II HLA-binding peptides range in length from 12 to 25 amino acids, and longer peptides may interfere with HLA binding, shorter versions of the impurity peptides were designed, featuring the HLA-binding region centered in the middle of the synthesized peptide.

#### Peptides for *in vitro* immunogenicity protocol naïve T-cell assays

SCT impurities featuring TCR-facing residue modifications and alterations in the overall number of T-cell epitopes as compared to the API were tested using T-cell assays. This method was used to assess the impact of the modification on T-cell responses, relative to baseline. Full-length peptides (both API and impurities) were evaluated in the T-cell assays.

Impurity peptides were synthesized by 21^st^ Century Biochemicals (Marlborough, MA). Their molecular weight was verified by mass spectrometry, and all peptides were determined to have a purity of higher than 95% by high-performance liquid chromatography (HPLC). The synthetic salmon calcitonin API peptide was provided by the Office of Generic Drugs. Miacalcin^®^ was purchased from Pharmaceutical Buyers Inc. (New Hyde Park, NY).

### Class II HLA binding assays

The class II HLA binding assays performed for these studies measure the binding affinity of a target peptide to HLA DRB1*0101, *0301, *0401, *0701, *0901, *1101, *1301, and *1501 in a standardized competition assay. This assay was originally described by Steere et al. (2006) and adapted by EpiVax. Previous publications have described the assay in detail (McMurry et al., 2007; Ardito et al., 2011). Briefly, unlabeled test peptides are incubated overnight to equilibrium with a soluble HLA DR molecule (Benaroya Research Institute, Seattle, Washington) and a biotinylated, allele-specific competitor peptide. The binding reaction is then neutralized, and peptide–HLA complexes are transferred to a 96-well plate coated with the pan-HLA DR antibody, clone L243 (BioLegend), and incubated overnight. The following day, Europium-labeled streptavidin (PerkinElmer, Waltham, MA) is added to identify the peptide–HLA complexes. An indirect measure of binding is determined by time-resolved fluorescence. Each peptide–HLA binding reaction is evaluated in triplicate over a range of seven concentrations. The percent inhibition values at seven distinct concentrations are used to calculate the IC_50_ value, defined as the concentration at which the test peptide inhibits 50% of the labeled competitor peptide.

Peptides that bind to the HLA at an IC_50_ value of 100 nM or less are considered very high-affinity binders. Peptides that bind at IC_50_ values ranging from 100 nM to 1,000 nM are considered high-affinity binders. Peptides that bind at IC_50_ values ranging from 1,000 nM to 10,000 nM are considered moderate-affinity binders. Peptides that bind at IC_50_ values ranging from 10,000 nM to 100,000 nM are considered low-affinity binders, and those with IC_50_ values ranging from 100,000 nM to 1,000,000 nM are considered negligible-affinity binders. Peptides that bind at IC_50_ values greater than 1,000,000 nM and those that do not exhibit dose-dependent inhibition of the competitor peptide are considered non-binders. We note that these concentrations are specific for the *in vitro* HLA-binding assay and are used to compare the relative binding affinities of the impurity sequences to the API and independent from the peptide concentrations described for the *in vitro* T-cell assays described below. A more detailed description of this assay, as used for generic peptide studies, and references to other comparable approaches is provided in Drug Discovery Today by De Groot et al. (2023).

### Human peripheral blood mononuclear cells

To improve the sensitivity of the T-cell assays, fresh (not frozen) blood samples were obtained from human blood donors for the T-cell assays (internal observations). Specifically, PBMCs were isolated from leukocyte reduction filters obtained from the Rhode Island Blood Center (RIBC) in Providence, RI. To confirm the sufficient breadth of HLA coverage in the donor cohort, high-resolution (4-digit) class II HLA haplotyping of donors was performed at the Transplant Immunology Laboratory at Hartford Hospital in Hartford, CT, using the sequence-specific oligonucleotide method (SSP-PCR). Five supertype alleles were expressed by the donor PBMCs used in the assays reported here (HLA DRB1*0301, *0401, *0701, *1101, *1301, and *1501). The HLA identified for the cohort of subjects used in this study is representative of 81.81% of the HLA alleles expressed by the global population. Forty percent of the donors were female and 60% of the donors were male, and their ages ranged from 17 to 83 years. Donor cohort demographic information and HLA types for each donor are provided in Supplementary Figure S1.

### *In vitro* immunogenicity protocol

To measure the naïve T-cell response to salmon calcitonin and impurity peptides, cells were plated at a density of 2.5 × 10^5^ cells per well in 96-well U-bottom cell plates in RPMI 1640 cell culture medium supplemented with IL-2 (10 ng/mL) and IL-7 (20 ng/mL) (Gibco). The cells were incubated with Miacalcin^®^, or a synthetic salmon calcitonin peptide, or individual peptide impurities, at a concentration of 20 μg/mL for 14 days at 37°C/5% CO_2_, with medium exchanges that include cytokine support (IL-2 and IL-7) on days 4, 7, and 11. A concentration of 20 μg/mL (5.8 µM) was the dose selected for the SCT API and the four selected impurities, following a dose-ranging study performed with Miacalcin^®^ to determine the maximum dose at which a T-cell response could be observed with no observed toxicity to the cells. The dose-ranging study was performed following the same protocol as described above.

The following control antigens were used in the T-cell assays for each of the PBMC samples: positive controls, keyhole limpet hemocyanin (KLH; Thermo Fisher), and the antigenic peptide pool CEFT (ImmunoSpot). In addition, human serum albumin (HSA; Sigma-Aldrich) was used as a negative control antigen, and phytohemagglutinin (PHA; Thermo Fisher), a T-cell mitogen, was used to confirm T-cell functionality. Donors for which a positive response to KLH, CEFT, and PHA and a negative response to HSA were observed were included in the final data compilation; donors that did not respond as expected to the control antigens were excluded. Responses were considered positive if (1) the number of spots was at least twice the background (stimulation index [SI] ≥ 2); (2) there were greater than 50 spot-forming cells (SFCs) per 1.0 × 10^6^ PBMCs; and (3) responses were statistically different from the media (Student’s *t*-test). Each of the test articles (SCT API, RLD, or impurity peptide) and each of the positive and negative controls were evaluated in nine replicate wells containing donor PBMCs.

### FluoroSpot assay

Following the 14-day incubation, cells in the nine replicate wells were collected, pooled by the treatment condition, and re-plated in triplicate on a precoated anti-human IFNγ FluoroSpot plate (Mabtech) at a concentration of 1.0 × 10^5^ cells per well in the presence of an appropriate test article or control reagent in supplemented RPMI 1640 culture medium. The FluoroSpot plates were incubated for 48 h at 37°C/5% CO_2_. After 48 h, FluoroSpot plates were developed according to the manufacturer’s directions. Spots were counted on an iSpot Spectrum FluoroSpot reading system (AID, Strassberg, Germany) using software 7.0, build 14790. Fluorophore-specific spot parameters were defined using the spot size, spot intensity, and spot gradient. A spot separation algorithm was applied for optimal spot detection. Analysis and counting of spots were performed by unbiased experts at ZellNet Consulting, Inc. (Fort Lee, NJ).

The IFNγ cytokine was selected as the biomarker for this assay. This cytokine supports the proliferation of early activated T cells and can induce Bcl-6 expression to drive follicular T-helper cell differentiation (Sweet et al., 2012). Bcl-6 is the signature transcription factor of follicular T-helper cells that are pivotal to promoting B-cell differentiation into high-affinity antibody-producing plasma cells and memory B cells. IFNγ also supports antigen presentation to CD4 T cells by increasing the quantity of peptide–HLA class II complexes on the surface of antigen-presenting cells through upregulated production of the HLA complex itself, the invariant chain, lysosomal proteases implicated in the peptide production for MHC loading (cathepsins B, H, and L), DMA and DMB (which remove CLIP from the class II HLA–peptide-binding cleft to make it available for peptide loading), and class II transactivator, a key transcription factor for the regulation of expression of genes involved in the class II HLA complex (Figueiredo et al., 1989; Chang and Flavell, 1995; Laha et al., 1995).

## Results

### *In silico* results

#### *In silico* analysis of the salmon calcitonin API sequence

SCT features an N-terminal 7-amino acid ring structure held in place by a disulfide bond between the cysteine in position 1 and the cysteine in position 7. As noted previously, HLA molecules bind to linear peptides. Before potential T-cell epitopes can bind to HLA molecules, secondary and tertiary peptide structures must be degraded. In this case, the disulfide bond between positions 1 and 7 may be reduced by gamma-interferon-inducible lysosomal thiol (GILT) reductase in the endocytic compartment (Hastings, 2013). For the purposes of *in silico* analysis, the disulfide bond was ignored, and the fully linearized sequence was analyzed. However, we note that the presence of disulfide bonds has been shown to impact human T-cell recognition (Mannering et al., 2005). Furthermore, the C-terminus of the SCT peptide is amidated in the drug product. This feature is not expected to impact the interaction between the peptide side chains of the SCT molecule and the binding pockets of any HLA molecule. Therefore, this feature was not considered in the *in silico* analysis.

A detailed *in silico* analysis of the salmon calcitonin API sequence as performed by EpiMatrix is shown in Figure 2 (see the figure legend for details on the interpretation of the assessment). As shown in the figure, the salmon calcitonin API sequence contains 12 predicted HLA DR ligands defined by EpiMatrix, which is slightly more than would be expected in a random peptide sequence of equivalent length. Seven of the 12 EpiMatrix-defined ligands are found within a single 9-mer frame of the SCT sequence located in frame 16. This feature indicates that the frame may serve as a promiscuous T-cell epitope and is likely to be immunogenic, especially as it contains relatively high-scoring HLA ligands, as defined using EpiMatrix. When considered in isolation, the frame 16 EpiBar has an EpiMatrix score of 14.26, which further indicates a high potential for immunogenicity, similar to other immunogenic T-cell epitope sequences. Additional EpiMatrix-defined ligands that may also be immunogenic are located in frames 8, 11, and 12—these sequences may be true ligands, but their effects may be HLA-restricted.

The JanusMatrix algorithm was then applied to the putative T-cell epitopes to estimate the cross-conservation of the epitopes with similar HLA-binding epitopes found in the human proteome. The salmon calcitonin API peptide has a low Human Homology Score (0.92), indicating that the putative epitopes found within the sequence are relatively unique to salmon calcitonin when compared to the human proteome.

To summarize the *in silico* results for the SCT API peptide, it contains slightly more epitopes than would be expected in a randomly generated peptide sequence of similar length, as identified using EpiMatrix. Considering its foreign origin, low JanusMatrix Human Homology Score, and the presence of multiple putative HLA ligands, the potential of the SCT API to drive a T-cell-dependent immune response was found to be slightly above average. Furthermore, the promiscuous T-cell epitope in frame 16 and the EpiMatrix-defined HLA ligands present in frames 8, 11, and 12 were considered likely to be contributing factors to the development of the anti-SCT immune response.

#### *In silico* analysis of SCT impurities

**FIGURE 3:**
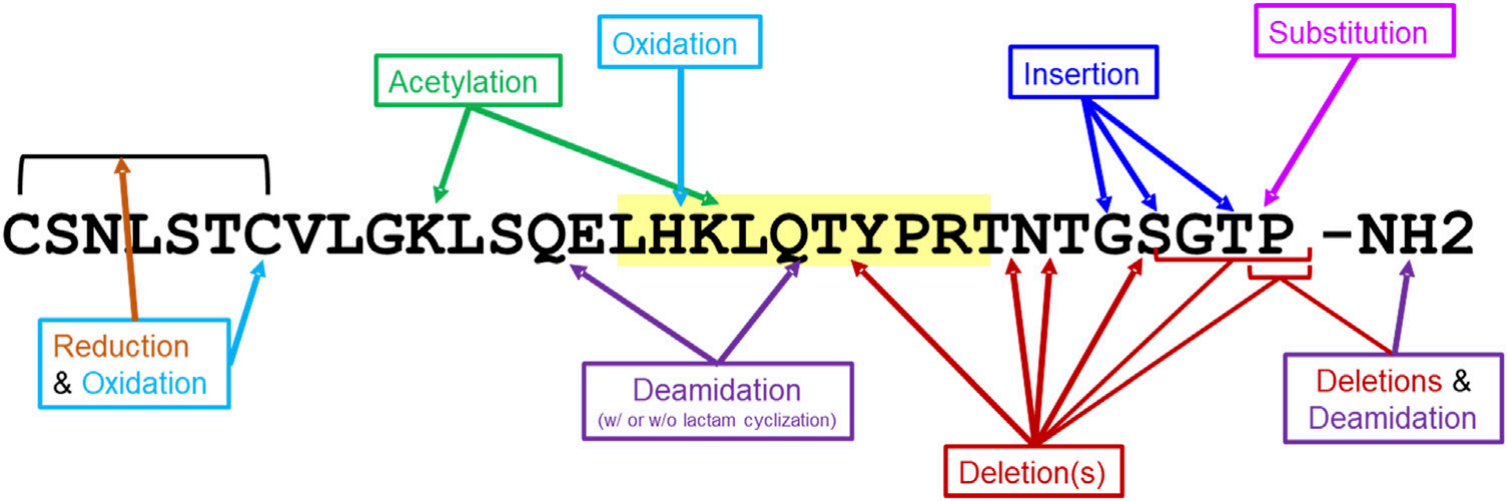
Impurity modifications on salmon calcitonin sequence. Twenty salmon calcitonin impurity peptides were analyzed for immunogenic potential *in silico*. The modifications to the salmon calcitonin active pharmaceutical ingredient (API) sequence are shown. The frame 16 promiscuous T-cell epitope is highlighted in yellow.

The 20 salmon calcitonin impurity sequences (Supplementary Table S2) were analyzed for T-cell-dependent immunogenic potential using EpiMatrix and JanusMatrix, as was described above for the SCT API. These 20 sequences include impurities that result from amino acid deletions and insertions, oxidation, acetylation, deamidation, and substitution (Figure 3). In addition to EpiMatrix and JanusMatrix analyses, these 20 peptide-related impurity sequences were also analyzed for new T-cell epitope content compared to the SCT API sequence.

The EpiMatrix immunogenicity scores of the submitted impurity sequences fall within the upper neutral range on the peptide immunogenicity scale, but above the score for most human proteins (including the human proteome and secretome), indicating potential for immunogenicity. The impurity scores were not significantly different from the EpiMatrix score of the SCT API across the 20 peptide-related impurities. In addition, a JanusMatrix analysis of the impurity sequences demonstrated scores ranging from 0.13 to 1.44. Like the parent API sequence, these lower JanusMatrix scores suggest limited potential for homology-induced tolerance. Relative to the SCT API, some of the impurity modifications create new epitopes. It was hypothesized that these new epitopes would be the most likely drivers of any potential impurity-induced immune responses.

**FIGURE 4:**
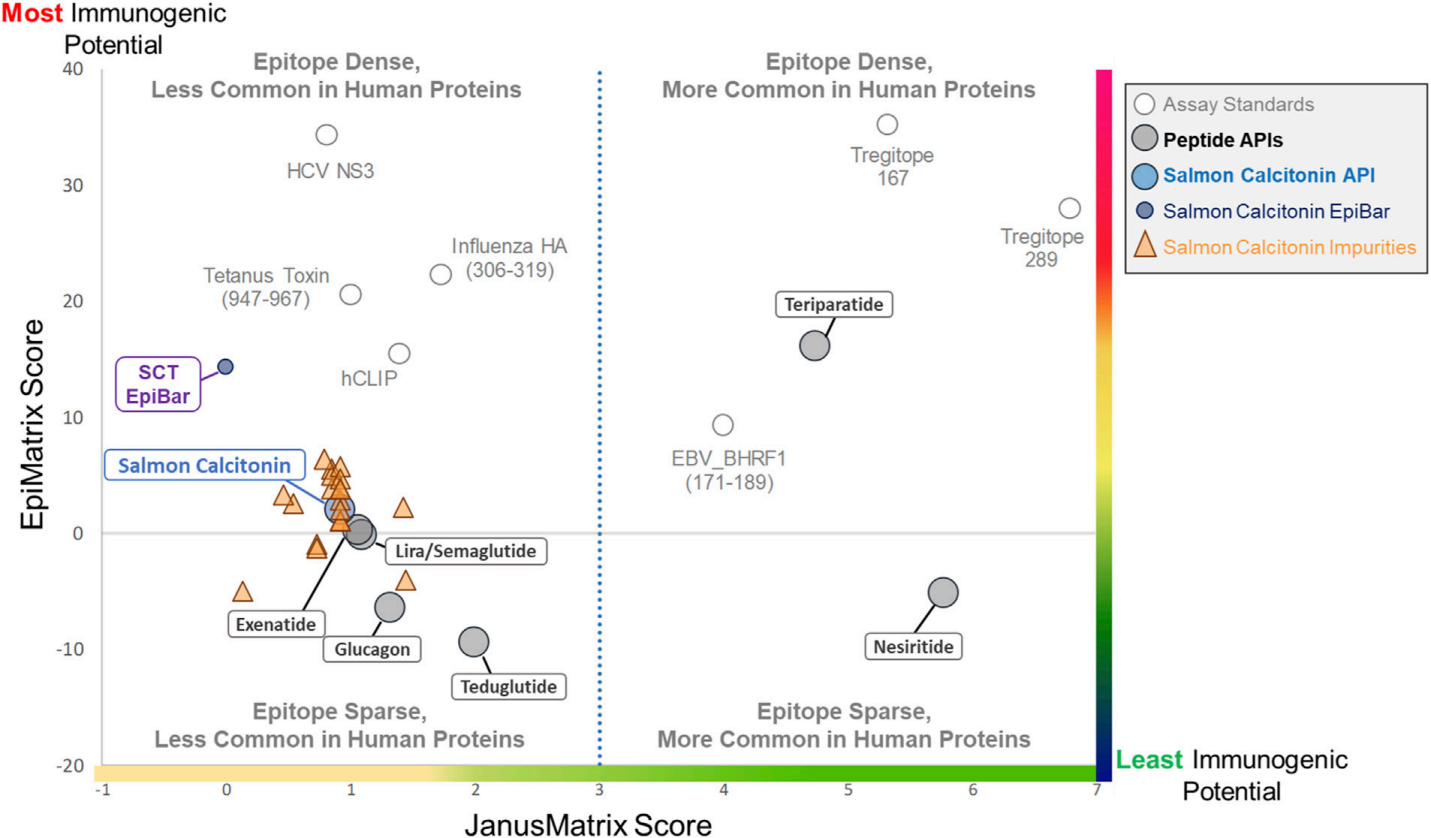
Immunogenicity quadrant plot. Immunogenicity quadrant analysis categorizes peptides and impurities by their immunogenicity risk. EpiMatrix (EMX) and JanusMatrix (JMX) human homology scores are plotted for each peptide and impurity. The graph is divided into four quadrants based on epitope content and human cross-conservation. Peptides and impurities that fall into the epitope-dense, less common in the human proteins quadrant are likely to induce an immune response, while peptides and impurities that fall into the epitope-sparse, more common in human proteins quadrant are considered a lower risk for inducing an immune response. Full-length salmon calcitonin scores above the expectation for a random peptide sequence (gray line), along with the full-length SCT impurities, falls into the epitope-sparse, less common in human proteins quadrant, which still carries some risk of immunogenicity. When assessed in isolation, the salmon calcitonin EpiBar falls into the epitope-dense, less common in human proteins quadrant. The EpiMatrix and JanusMatrix scores of several “benchmark” peptides are included to aid in the interpretation of relative risk.

The results of the *in silico* analysis of the SCT API and 20 related impurities are shown in an immunogenicity quadrant plot (Figure 4). This figure shows that peptides and their impurities can be classified into one of four quadrants defined as putative T-cell epitope-dense or epitope-sparse, more common in human proteins and less common in human proteins. Immunogenic (vaccine epitope) and tolerogenic (Treg epitope) peptides are included as examples or “control” peptides in the relevant quadrants.

Salmon calcitonin and its impurities fall into the “Epitope Sparse, Less Common in Human Proteins” quadrant. Peptides in this quadrant still carry some risk of immunogenicity, particularly peptides containing foreign T-cell epitopes like salmon calcitonin and most of the analyzed impurity sequences. The overall score of the promiscuous T-cell epitope in the peptide (see SCT EpiBar, in Figure 4) is much higher and more foreign than that of most generic peptides. Thus, much of the observed immunogenicity to SCT is attributed to foreign epitopes within the API sequence.

#### Class II HLA binding studies

To determine the impact of modifications to HLA-facing amino acid residues on HLA binding, 3 of the 20 identified impurities, LYS-AC11 (acetylation of lysine 11), DES-THR21 (deletion of threonine 21), and DES-ASN26 (deletion of asparagine 26), were selected for *in vitro* validation in the class II HLA-binding assay (Table 1). These three impurities were selected based on the *in silico* analysis for modifications to HLA-facing residues of the predicted epitopes. For the *in vitro* HLA-binding assays, the salmon calcitonin API was divided into N- and C-terminal peptides, which permitted a more accurate evaluation of the impact of the impurity modifications on HLA binding relative to the corresponding region of the API peptide as longer peptides tend to take on secondary structures *in vitro*, which may interfere with accurate binding assessments. These shorter versions of the API and impurity peptides were evaluated for their ability to bind to a panel of eight class II HLA DRB1 supertype alleles (Southwood et al., 1998), namely, HLA DRB1*0101, *0301, *0401, *0701, *0901, *1101, *1301, and *1501.

**TABLE 1:** Sequence of salmon calcitonin and selected impurities evaluated in the class II human leukocyte antigen (HLA)-binding assay.

Peptide name	HLA-binding assay peptide sequence	EMX hits	EMX score	JMX score
API: salmon calcitonin	*N-term:* ST S VLGKLSQELHKLQTY	12	1.99	0.92
	*C-term:* SQELHKLQTYPRTNT			
IMP: LYS-AC11_SCT	ST S VLG ( Ac-K )LSQELHKL	13	5.55	0.85
IMP: DES-THR21_SCT	SQELHKLQ - YPRTNTGSGT	9	−4.06	1.44
IMP: DES-ASN26_SCT	HKLQTYPRT - TGSGT	14	6.38	0.79

Short peptides compatible with the HLA-binding assay were designed to center the predicted epitope content for the API and three impurities. Note that in peptides synthesized for the HLA-binding assays, the cysteine in position 7 was replaced with serine (shown in blue) with no predicted impact on binding relative to the native peptide. This substitution was made to prevent the formation of aggregates due to disulfide bond formation between cysteines in the peptides. EpiMatrix (EMX) hits are the number of predicted HLA ligands in the full-length sequence. The EpiMatrix (EMX) score indicates the score for the full-length sequence. JanusMatrix (JMX) homology scores indicate the average depth of epitope cross-conservation with the human proteome. For a foreign peptide, JMX scores above 2.00 are considered to have an elevated potential for homology-induced tolerance. Residues in red indicate differences in the impurity sequence as compared to the API; for the deletion impurities (DES) a red (-) represents the deleted amino acid.

**FIGURE 5:**
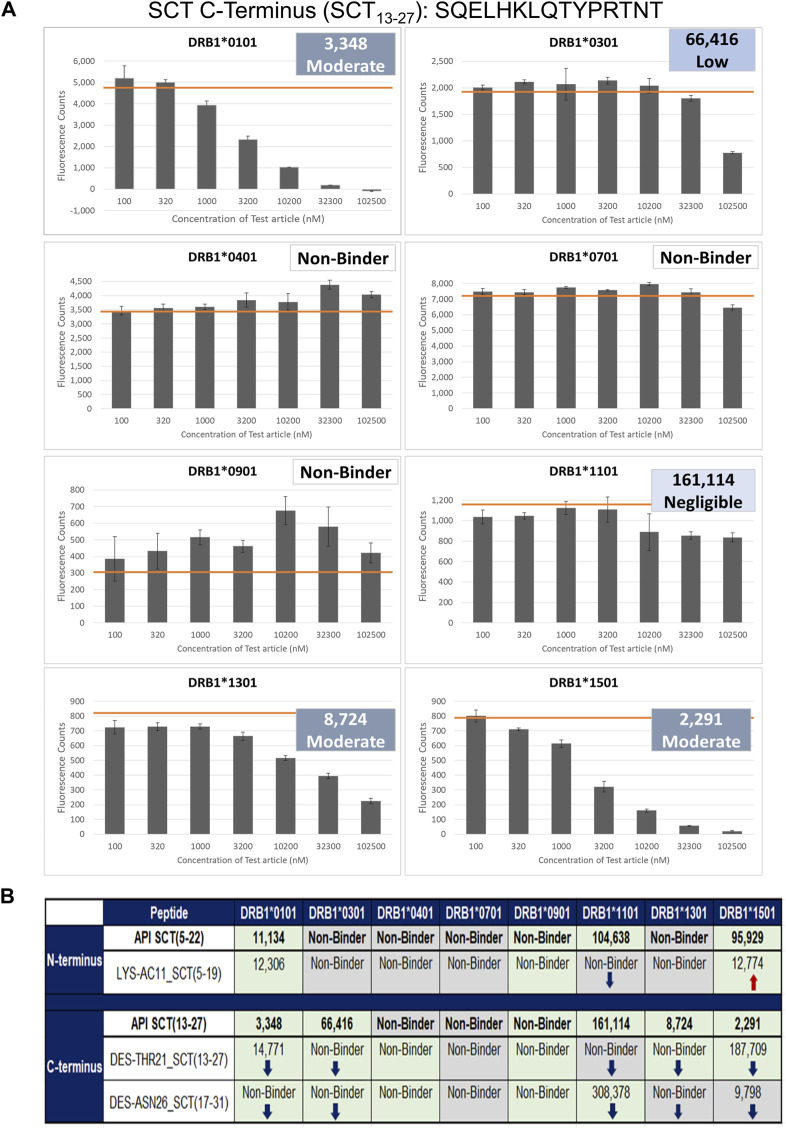
Results of the class II HLA-binding assay. **(A)** Results of a typical assay shown for the C-terminal API SCT (13–27) peptide. The orange bar on each graph denotes the maximum fluorescence value (no inhibition). **(B)** Summary of the binding results for the three impurities listed in Table 1. Top half of the table: results for the N-terminal SCT API peptide and its impurity. Bottom half of the table: the C-terminal peptide and its two impurities. Green-shaded box: binding results align with *in silico* analysis results; no change in binding was expected or observed. ↓Blue arrows: binding is decreased, consistent with *in silico* assessment results. ↑Red arrows: binding is increased, consistent with *in silico* assessment results. Gray-shaded box: Binding diverges from *in silico* assessment.

These two API and three SCT impurity peptides (LYS-AC11_SCT, DES-THR21_SCT, and DES-ASN26_SCT) were evaluated for binding in HLA-binding assays (Table 1). As previously mentioned, these impurities had been selected for binding assays because of observed modifications to HLA-facing residues, suggesting that they may bind differently from the API, impacting the T-cell response. The N-terminal API peptide serves as the HLA-binding control for the LYS-AC11_SCT impurity, whereas the C-terminal API peptide provides the baseline HLA-binding control for impurities DES-THR21_SCT and DES-ASN26_SCT. The results of the HLA-binding assays for the SCT C- and N- terminal peptides were generally aligned with the results of the *in silico* analysis of salmon calcitonin. For example, the N-terminus of SCT, which shares the greatest sequence homology with human calcitonin and contains limited T-cell epitope content compared to the C-terminus, did not bind to HLA DR with high affinity, which is consistent with the *in silico* analysis result. As expected, the N-terminal peptide STSVLGKLSQELHKLQTY bound to supertype allele HLA DRB1*1101 with negligible affinity (IC_50_ = 104,638 nM) and to alleles HLA DR*0101 (IC_50_ = 11,134 nM) and *1501 (IC_50_ = 95,929 nM) with weak affinity (Figure 5). These results indicate that the N-terminus of SCT is not expected to contribute to the immunogenicity of SCT due to minimal T-cell epitope content and its weak binding profile in these assays.

In contrast, the C-terminal salmon calcitonin API peptide, SQELHKLQTYPRTNT, containing the majority of the putative foreign T-cell epitopes, bound five of the eight HLA alleles evaluated in these assays. These results align with the *in silico* identification of a C-terminal EpiBar and with the results published by Kozono et al. (1992) and Tangri et al. (2005). The C-terminal peptide bound with moderate affinity to DRB1*0101 (IC_50_ = 3,348), *1301 (IC_50_ = 8,742), and *1501 (IC_50_ = 2,291) and to DRB1*0301 with weak affinity (IC_50_ = 66,416). It bound with negligible affinity to DRB1*1101 (IC_50_ = 161,114) (Figure 5).

#### Results of impurity peptide binding studies

##### LYS-AC11_SCT

LYS-AC11_SCT is an N-terminal SCT impurity, where the lysine in position 11 is acetylated relative to the baseline sequence. The sequence of this impurity peptide is STSVLG (Ac-K)LSQELHKL. The *in silico* analysis was aligned with the following results: LYS-AC11_SCT bound with weak affinity to the DRB1*0101 allele (IC_50_ = 12,306 nM) and to the DRB1*1501 allele (IC_50_ = 12,774 nM). Compared to the N-terminal API peptide, binding was nearly equivalent for DRB1*0101. LYS-AC11_SCT showed increased affinity for the DRB1*1501 allele. Similar to the API control peptide, LYS-AC11_SCT is a non-binder to DRB1*0301, *0401, *0701, *0901, and *1301. Relative to the SCT API peptide, a loss of binding to DRB1*1101 was observed for LYS-AC11_SCT. Consistent with our expectations based on the *in silico* analysis, LYS-AC11_SCT exhibited an increase in binding affinity to the DRB1*1501 allele, and no change in binding was observed for DRB1*0101 and *0901. In contrast to what we expected from the *in silico* analysis, no change in binding affinity was observed for alleles HLA DRB1*0301, *0401, *0701, *1101, and *1301.

##### DES-THR21_SCT

DES-THR21_SCT is a C-terminal SCT impurity, where the threonine in position 21 is deleted relative to the baseline sequence. The sequence of this impurity peptide is SQELHKLQ–YPRTNTGSGT. *In silico* analysis was aligned with *in vitro* observations: EpiMatrix analysis indicated that the SQELHKLQ–YPRTNTGSGT would no longer bind to the DRB1*0301, *0401, and *1301 alleles *in vitro*. Consistent with the *in silico* assessment results, DES-THR21_SCT did not bind to any of these three alleles. Further in alignment with the *in silico* analysis, DES–THR21_SCT bound to both DRB1*0101 (IC_50_ = 14,771 nM) and *1501 (IC_50_ = 178,709 nM) with reduced affinity as compared to the C-terminal API peptide. Consistent with the *in silico* analysis results, DES-THR21 showed decreased affinity to HLA DRB1*1301, and no change in binding was observed for alleles DRB1*0101, *0301, *0401, *0901, and *1501. Contrary to the *in silico* analysis, no change in binding affinity was observed for HLA DRB1*0701, and a decrease in binding affinity was observed for HLA DRB1*1101.

##### DES-ASN26_SCT

DES-ASN26_SCT is a C-terminal SCT impurity, where the asparagine in position 26 is deleted relative to the baseline sequence. The sequence of this impurity peptide is HKLQTYPRT–TGSGT. This amino acid deletion is located outside of the frame 16 EpiBar, in a region of the peptide where minimal epitope content was identified *in silico*. Relative to the SCT C-terminal API peptide, the DES-ASN26_SCT impurity was expected to show a loss of binding to DRB1*0401 but to gain affinity to DRB1*1301. This impurity peptide bound with negligible affinity to DRB1*1101 (IC_50_ = 308,378 nM) and with moderate affinity to DRB1*1501 (IC_50_ = 9,789 nM). Consistent with the *in silico* assessment, no binding was observed based on the *in silico* analysis, no binding was observed for alleles HLA DRB1*0101, *0301, *0401, and *0901, and binding affinity was decreased for DRB1*1101. In contrast to our assumptions based on the *in silico* analysis, there was no change in binding affinity to HLA DRB1*0701 and *1501.

#### Evaluation of salmon calcitonin and impurities in *in vitro* naive T-cell assays

To evaluate the impact of peptide impurities present within the generic SCT product on its overall immunogenic potential, two separate approaches were employed. In the first study, the SCT API and several individual peptide impurities were evaluated at equivalent concentrations in *in vitro* T-cell assays to determine their individual immunogenic risk potential (n = 16 donors). In the second study, the RLD product, Miacalcin^®^, was spiked with individual impurities equal to the observed abundances identified in the generic SCT product, and the combination was tested in T-cell assays. The drug product assay was designed to estimate the ability of the impurities to increase the immunogenic potential relative to the drug product alone (n = 20 donors).

Four SCT impurities were selected for validation using T-cell assays based on changes to TCR-facing residues as these were expected to modify the immunogenic potential of each peptide relative to the API *in vitro*: LYS-AC18 (acetylation of lysine 18), Q20E_SCT (glutamine in position 20 is deamidated to form glutamic acid), ENDO-GLY28 (insertion of glycine at position 28), and ENDO-THR31_SCT (insertion of threonine at position 31). Peptide sequences, EpiMatrix scores, and JanusMatrix scores are listed in Table 2.

**TABLE 2:** Peptides evaluated using T-cell assays.

Peptide name	HLA-binding assay peptide sequence	EMX hits	EMX score	JMX score	Relative abundance (% of API)
API: salmon calcitonin	CSNLSTCVLGKLSQELHKLQTYPRTNTGSGTP	12	1.99	0.92	n/a
IMP: LYS-AC18_SCT	CSNLSTCVLGKLSQELH (Ac-K) LQTYPRTNTGSGTP	13	3.33	0.46	0.24%
IMP: Q20E_SCT	CSNLSTCVLGKLSQELHKLETYPRTNTGSGTP	11	−0.92	0.73	0.36%
IMP: ENDO-GLY28_SCT	CSNLSTCVLGKLSQELHKLQTYPRTNT GG SGTP	12	1.06	0.92	2.62%
IMP: ENDO-THR31_SCT	CSNLSTCVLGKLSQELHKLQTYPRTNTGSG TT P	12	1.06	0.92	3.30%

Peptides were selected based on changes to the TCR-facing residues of the predicted epitopes. The observed relative abundance of the impurities was provided as the percentage of the API by the FDA. Amino acid modifications to the impurity peptides are in red. Full-length peptides synthesized for the IVIP T-cell assays contain a disulfide bond between the cysteines in positions 1 and 7, forming the N-terminal ring structure present in the native salmon calcitonin peptide. API, active pharmaceutical ingredient; IMP, abbreviate impurity. EpiMatrix (EMX) hits are the number of predicted HLA ligands in the full-length sequence. JanusMatrix (JMX) homology scores indicate the average depth of epitope cross-conservation with the human proteome. JMX scores above 2.00 are elevated for foreign-derived sequences and indicate potential for homology-induced tolerance. Residues in red indicate differences in the impurity sequence as compared to the API.

Of the blood donors selected for this cohort, 40% were women, and 60% were men. The ages of donor cohort members ranged from 17 to 83 years. The following class II HLA supertype alleles were expressed by the donor PBMCs used in these assays: HLA DRB1*0301, *0401, *0701, *1101, *1301, and *1501 (see Supplementary Figure S1 for detailed HLA information about each donor). No differences in the naïve T-cell response rate were related to the age or sex of the donor in this dataset (data not shown).

**FIGURE 6:**
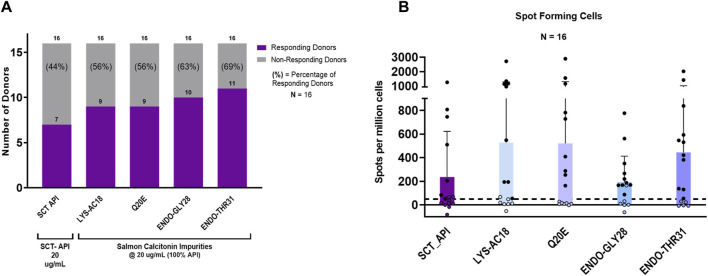
IVIP immunogenic potential. **(A)** Comparison of the number of donor PBMCs responding to the salmon calcitonin (API) *versus* each impurity. Donor PBMC positivity includes three criteria: (1) INFg spot-forming cells (SFCs) > 50, (2) stimulation index (SI) > 2, and (3) a statistical difference between medium and peptide stimulation as determined by Student’s *t*-test (*p* < 0.05). The *left panel* shows the number (purple bar) and percentage of responders (%) to each of the test peptides. All peptides were evaluated at 20 μg/mL, and the total number of donors evaluated was 16. **(B)** The r*ight panel* shows the comparison of individual donor responses by the number of IFNg SFCs for the SCT API and peptide impurities. Note that the mean response for impurity Endo-GLY28 is lower than the mean response for the SCT API and other impurities, but 10 donor PBMC samples met the positivity criteria described. Filled circles indicate responsive donors that met the positivity criteria described above. The dashed line indicates the SFC positivity threshold at 50 SFCs.

For the initial set of assays, the peptides were each evaluated at a concentration of 20.0 µg/mL in 16 individual donor PBMC samples. As shown in Figure 6A, the salmon calcitonin API peptide elicited a response in 44% (7 of 16) of the donors. This high overall response is consistent with the clinical observations and the *in silico* analysis results.

Interestingly, of the 10 that showed no response to the API, 7 responded to 1 or more impurities. Indeed, compared to the salmon calcitonin API at an equivalent concentration (20 μg/mL) *in vitro*, LYS18_SCT (5.8 µM) and Q20E_SCT (5.8 µM) generated a response in 56% (9 of 16) of donors, ENDO-GLY28_SCT (5.7 µM) in 63% (10 of 16) of donors, and ENDO_THR31_SCT (5.7 µM) in 69% (11 of 16) of donors (Figure 6A) (see Supplementary Table S3 for peptide-specific responses by donors). A substantial difference between the number of responding PBMC donors to the API and the four impurities was not observed; however, this may be due to the limited sample size. Differences in PBMC donor responses between the API and the impurities shown in Figure 6A were more striking when the results were normalized to the results for the salmon calcitonin API peptide (Supplementary Figure S2A). These results indicate that both the SCT API and selected impurities evoke immune responses from a high proportion of PBMC donors when tested *in vitro*. These results suggest that individually, the API and impurities have the potential to cause anti-drug immunity, although not necessarily in the same subjects.

Of note, the evaluation of a number of SFCs by each of the PBMC donor responses showed that although LYS-AC18 and Q20E elicited a higher number of IFNg-secreting cells in some donors, the differences in the intensity of the response between the API and individual impurities were not significant (Figure 6B). Two non-parametric tests, the Mann–Whitney and Wilcoxon matched-paired signed rank tests, were used to measure statistical differences in the SFC response between the API and the four impurities. For both tests, no statistical significance was observed for all comparisons.

Overall, these results suggest that several of the evaluated impurities have the potential to enhance the immunogenicity of the drug product when evaluated at the same concentration as the API. However, the observed difference in the number of donor samples that responded to the API and to the individual impurities was not sizable, and the difference in the intensity of response (SFCs) did not reach statistical significance (Mann–Whitney and Wilcoxon matched-paired rank tests).

**FIGURE 7:**
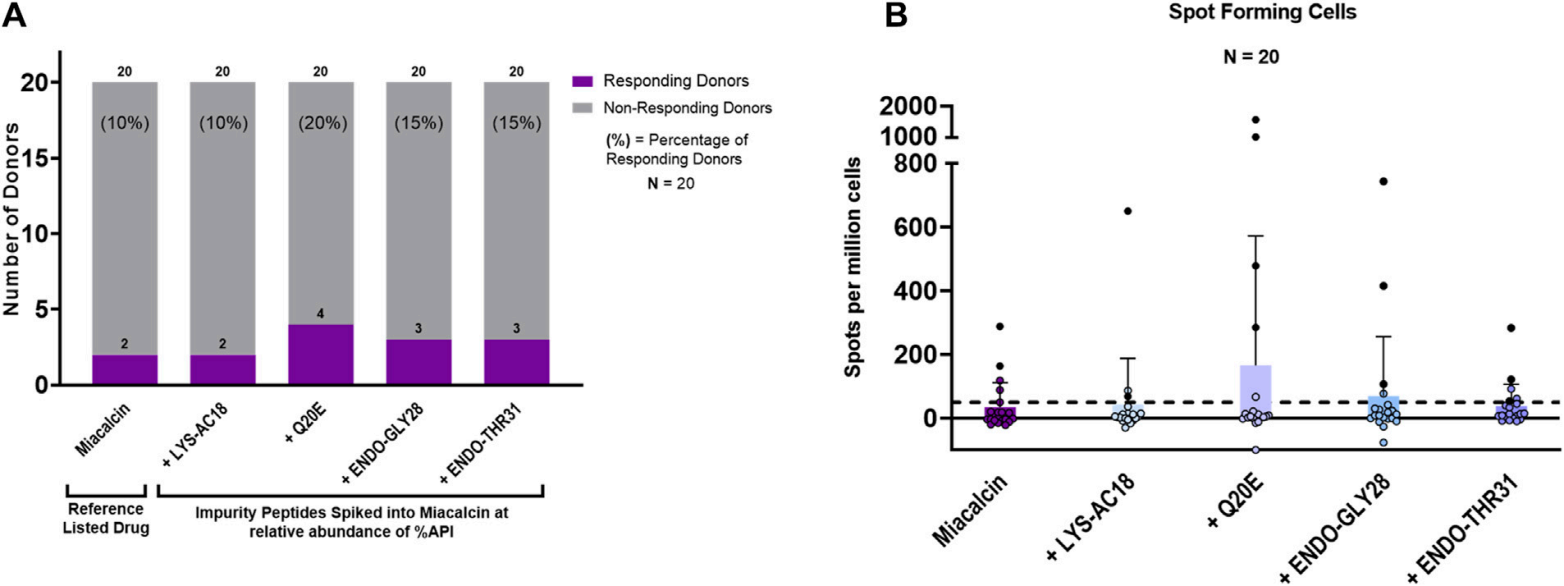
IVIP spiked impurities. **(A)** Comparison of the Miacalcin^®^ drug product to the Miacalcin spiked with individual impurities at their observed concentrations relative to the API. Donor PBMC positivity includes three criteria: (1) INFg SFCs > 50, (2) SI > 2, and (3) a statistical difference between medium and peptide stimulation as determined by Student’s *t*-test (*p* < 0.05). The *left panel* shows the number (purple bar) and percentage of responders (%) to each of the test peptides. The total number of donors tested was 20. Miacalcin was evaluated at 20 μg/mL. The LYS-AC18_SCT impurity was added at 0.24%, Q20E_SCT at 0.36%, ENDO-GLY28 at 2.62%, and ENDO-THR31_SCT at 3.30% of SCT in Miacalcin. **(B)** *Right panel* shows the comparison of individual donor responses by the number of IFNg SFCs for Miacalcin and Miacalcin spiked with peptide impurities. Black-filled circles indicate responsive donors that met the positivity criteria described above. The dashed line indicates the SFC positivity threshold at 50 SFCs.

Taking into consideration that the impurities, although immunogenic, would be present at a lower concentration than the API in an SCT preparation, we next sought to determine whether the impurities could enhance the immunogenic potential of SCT in the presence of the drug product. For these experiments, a second set of T-cell assays was performed in which the reference drug product, Miacalcin^®^, was “spiked” with the individual impurities at the concentrations at which they were observed in a generic SCT product provided by the FDA (Table 2). As shown in Figure 7A, three out of four impurities increased the overall incidence of response among PBMC donors when added to Miacalcin^®^ at their observed abundances when compared to Miacalcin^®^ alone. Differences in PBMC donor responses between the API and the impurities shown in Figure 7A were easier to observe when normalized to the results for the salmon calcitonin API peptide (Supplementary Figure S2B) for each of the donor PBMC results.

Of note, the overall percentage of donors responding to the Miacalcin^®^ drug product was much lower than that observed in the assays performed with the API peptide. This may have been due to the impact of the product formulation buffer on immune responses *in vitro* (Thacker et al., 2022). Despite the disparity between the results for the API and the formulated drug product (discussed in Supplementary Material: Impact of formulation on *in vitro* T-cell assays), these studies provide support for the hypothesis that product-related impurities can potentially increase the immunogenicity risk of generic drug products and suggest that the use of orthogonal methods to assess their binding to the MHC and their ability to induce novel T-cell responses may be useful in understanding the immunogenicity risk they pose.

Of the two PBMC donors that responded to Miacalcin^®^, only one responded to the Miacalcin^®^ spiked with the impurity peptides; specifically, this PBMC donor responded to the RLD product spiked with Q20E_SCT, ENDO-GLY28_SCT, or ENDO-THR31_SCT. Spot counts for Miacalcin^®^ spiked with impurity Q20E_SCT or ENDO-GLY28_SCT were higher than those for Miacalcin^®^ alone for this donor. Additionally, donors that responded to Miacalcin^®^ plus Q20E_SCT or ENDO-GLY28_SCT exhibited higher spot counts to the spiked product than the responses to Miacalcin^®^ alone, indicating a more robust response to the impurities (Supplementary Table S4).

In summary, SCT is an interesting peptide, given its significant immunogenic potential linked to the presence of multiple non-human HLA ligands. The above data suggest that impurities introduced during the manufacturing of the peptide could elicit responses from donors that do not respond to SCT, underscoring the need to identify and control these peptides to reduce the potential risk.

## Discussion

This study describes the application of *in silico* and *in vitro* methods to evaluate the immunogenicity risk potential of synthetic peptide impurities that could be present in generic versions of Miacalcin^®^. For this initial evaluation of orthogonal methods that could be used to assess immunogenicity risk, the SCT API and three synthesis-related impurity peptides identified in the synthetic SCT API were assessed for the presence of new HLA ligands (putative T-cell epitopes) in HLA-binding assays, and four impurities were compared to the API in independent T-cell assays.

Each generic product is likely to have different inherent immunogenic risk potential levels depending on the sequence of the peptide itself. In this case study, most of the putative T-cell epitopes identified in SCT (API sequence) and its impurities were unlike any similar ligand or epitope in the human genome. Regardless of the generic peptide drug, homologies between epitopes in the API and the human genome may influence the potential of the API peptide to induce immune responses, and therefore, homology between HLA-binding peptides in the API and the human proteome should be evaluated.

Consistent with its clinical history, the *in silico* analysis of SCT revealed that SCT contains multiple features that indicated a high potential for immunogenicity. These features include 1) a promiscuous 9-mer binding frame epitope bar, or “EpiBar,” located within the C-terminus of the peptide, and 2) minimal homology between the HLA ligands identified in the SCT API sequence and the human proteome. Due to high HLA binding potential and the non-self-nature of the region, this region of the peptide is likely responsible for the immunogenicity associated with salmon calcitonin, *in vivo*. This *in silico* analysis also confirmed published findings of a C-terminal immunogenic region (Kozono et al., 1992; Tangri et al., 2005). The results presented here further show that *in silico* analysis may be useful for identifying epitopes within peptide drugs that have the potential for eliciting unwanted immune responses and that the *in silico* analysis data may be useful when designing peptides comparing these regions to the corresponding regions of impurities.

We next compared the *in silico* and *in vitro* binding of SCT and selected impurities. Following *in silico* analysis, three impurities that showed changes to HLA-binding residues (positions 1, 4, 6, and 9 of a predicted binding frame), and could, therefore, impact HLA binding, were selected for class II HLA-binding assays. The *in silico* assessment results generally aligned with the results of class II HLA DRB1 *in vitro* binding assays. These assays demonstrated that the C-terminal region containing the promiscuous T-cell epitope, and impurities containing this same feature, bound to multiple class II HLA DRB1 alleles. The N-terminus of SCT and the impurity that included an amino acid side-chain modification in this region exhibited reduced HLA binding relative to the C-terminus. A review of published HLA-binding assays in the Immune Epitope Database (IEDB) (Tangri et al., 2005) (using overlapping 15-mer peptides) showed that the sequences in frames 8 (VLGKLSQEL) and 16 (LHKLQTYPRT) were reported to bind to several HLA DRB1 alleles with low to high affinity, confirming the results of both the *in silico* assessment and the HLA-binding assays reported here.

Some HLA-specific binding results for the impurities were not aligned with those of the *in silico* analysis. Differences between *in silico* and *in vitro* HLA-binding results are not unexpected for the N-terminal region impurity as the impurity featured unnatural or modified amino acids (Lys-Ac) for which accurate *in silico* models were not yet available. Because the *in silico* and *in vitro* results were not 100% aligned, this case study underscores the advantages of combining *in silico* analysis with *in vitro* testing to better assess the immunogenicity risk. This is especially true when unnatural amino acids or post-translational modifications are present in generic peptide impurities. The combination of two orthogonal methods is likely to provide a more accurate picture of overall immunogenicity risk. Additional research is warranted to elucidate the impact of post-translational modifications on immunogenicity risk.

The study also examined the relative risk of SCT and additional selected impurities using an *in vitro* naïve donor T-cell assay to determine their overall immunogenic risk potential. Two different strategies were employed: (1) T-cell responses to the individual peptide impurities were compared to the responses to a synthetic SCT peptide and (2) donor PBMC responses to the SCT RLD drug product (Miacalcin^®^) were compared to the RLD that had been spiked with the impurities.

Consistent with the results of the *in silico* analysis, the API was observed to be immunogenic, inducing immune responses in a large proportion of donor PBMC samples, *in vitro*. Importantly, the selected SCT variants induced immune responses from donors that did not respond to SCT when evaluated at an equivalent concentration.

Of note, the proportion of donor PBMC samples responding to impurities spiked into RLD Miacalcin^®^ was lower, but this could be due to the diminished immune response to SCT that was observed when using the Miacalcin^®^ drug product when compared to the API peptide. The presence of excipients in the drug product formulation may have impacted the health of the cells in culture (see Supplementary Materials for a discussion of the SCT formulation and its impact on immune response). This information is provided as a caution to future practitioners of the *in vitro* method of immunogenicity risk assessment. The use of the drug product formulation in *in vitro* assays may lead to inaccurate immunogenicity risk assessment due to poor cellular viability. It has been suggested that harmful excipients can be removed from the drug product by dialysis prior to their inclusion in *in vitro* studies. For the purpose of evaluating the immunogenicity of generic drug products, however, we believe that it is important to evaluate the intact product as it would be administered to the patient. Excipients within the formulation buffer that could be removed through dialysis may promote a separate innate immune response that could impact the overall adaptive immune response (Thacker et al., 2022).

The presence of pro-inflammatory cytokines and chemokines released by activated T cells targeting new epitopes can spread, inducing local and usually transitory autoimmune responses related to co-expressed “self” epitopes (Dalum et al., 1997; Powell and Black, 2001; Disis et al., 2004; Hintermann et al., 2011). In contrast, peptide epitopes that are identical in both the unmodified product and the amino acid sequences of product impurities (common epitopes) may engage and activate cognate T cells but are unlikely to induce new immune responses targeting the API. In summary, these *in vitro* assays provide an approximate indicator of the potential for an immune response under clinical conditions. In our study, the overall number of donor PBMC samples responding to the impurity peptides was increased, although the differences in responses between the API and the impurities did not reach statistical significance. This is likely to be due to the size of the cohort. Currently, the FDA recommends using at least 30 donors to achieve broad HLA coverage with multiple donors per allele. While broad HLA coverage can be achieved by including 30 donors for the *in vitro* T-cell assays, additional donors may aid in the interpretation of data for high-risk, low-frequency HLA alleles.

As demonstrated in this case study, when performing *in vitro* T-cell assays, the number of PBMC donors should be large enough 1) to provide power to the analysis and 2) to obtain sufficient breadth of global HLA coverage. Assays performed for regulatory submissions would ideally include at least two, if not three, donors per HLA type, and greater than 80% of the HLA alleles in the United States population should be represented. Currently, adequate HLA coverage can be attained by obtaining PBMCs from at least 30 blood donors. However, for peptide drugs that have low overall immunogenicity (as assessed by *in silico* analysis), it may be necessary to increase the number of donors further to confirm the low immunogenicity of the impurities in assays that are intended to confirm a negative response (ImmunXperts, Denies S. et al., manuscript in process).

Our study shows that the use of the three orthogonal approaches discussed here, together with an assessment of innate immune response-modulating impurities (Mattei et al., 2022), can provide information regarding the potential increase in risk immunogenicity due to impurities in the product. However, neither *in silico* nor *in vitro* assays recapitulate the complex interactions between the generic drug, generic drug impurities, and immune system components that occur *in vivo*. For example, the studies performed did not address HLAs other than HLA DRB1. Other HLAs may contribute to immune responses, although in retrospective and prospective studies, HLA DRB1 is most often associated with unwanted immune responses to biologics (Hyun et al., 2021; Ramarathinam and Purcell, 2021). In addition, the impact of unnatural amino acids on the accuracy of *in silico* and *in vitro* studies may be over or underestimated. Further research is necessary to improve the accuracy of the predictions.

The use of primary cells, such as PBMCs isolated from whole blood from human donors, can present its own set of challenges, including donor-to-donor variability in the observed T-cell responses *in vitro*. Such variability can be attributed to a host of factors including genetic differences at the individual donor level and the health of the individual at the time of donation, which is often self-reported. When performing a comprehensive immunogenicity risk assessment, however, it is important to consider the risk to the population as a whole rather than on an individual basis. Variability in the results of a risk assessment is expected when using primary cells; however, it is important to remember that the emphasis of the immunogenic risk is not on the individual donor but on the collective cohort. This approach of evaluating a broad range of individual donors provides an insight into the risk posed to the population.

Finally, regardless of how many different donor PBMC samples are included in *in vitro* assays, the complexity of the human HLA system precludes an accurate representation of the diverse immune responses that may occur in a human population. Furthermore, it is important not to overestimate the ability of *in vitro* assays to accurately replicate clinical conditions. For example, generic drug products may be administered repeatedly during treatment. Repeated dosing may further increase immunogenicity or, in contrast, could contribute to tolerance (depending on the nature of the immune response). Further investigation into the impact of repeated dosing on immunogenicity in clinical settings is warranted.

## Conclusion

This case study shows that the *in silico* analysis of the API and any new impurities provides a good starting point for evaluating the immunogenicity risk potential of a generic peptide. An initial *in silico* assessment of the immunogenic risk potential posed by peptide impurities may enable manufacturers to adjust their processes to remove the highest-risk impurities prior to initiating *in vitro* studies. Once the final impurities are identified, *in silico* assessments of potential HLA binding can then be validated with HLA-binding assays and/or T-cell assays. The selection of the type of assay to be performed and the need to query specific HLA types may depend on the findings in the initial *in silico* analysis. Immunogenicity risk assessment accuracy is improved by the use of orthogonal techniques to better assess the relative risk contributed by the impurities in the ANDA product.

## Figures and Tables

**Table:** 

**ADA**	anti-drug antibody
**ANDA**	abbreviated new drug application: drug application to the U.S. Food and Drug Administration to manufacture and market a generic drug in the U.S. The application is termed “abbreviated” since it does not require preclinical (animal) and clinical trials (human) to show safety efficacy but requires the applicant to demonstrate bio-equivalence to the reference listed drug (RLD)
**API**	active pharmaceutical ingredient: this is the active ingredient in a drug product
**APC**	antigen presenting cell: a type of immune cell (e.g. dendritic cell, macrophage and B cell) that binds peptide antigens via major histocompatibility complex (MHC) proteins on its surface and presents them to T cells
**Contaminant**	This include extractables and leachables and other potential impurities derived from the manufacturing process. These impurities are not related to peptide
**DP**	drug product: The finished dosage form of a drug that includes the active pharmaceutical ingredient and the inactive ingredients
**Formulation**	The process of the chemical and API components into the final drug product
**HLA**	human leukocyte antigen: also referred to as MHC, major histocompatibility complex, a group of highly polymorphic proteins that display peptide antigens to T cells
**IVIP**	*in vitro* immunogenicity protocol: an *in vitro* assay that is sufficiently prolonged to measure the expansion and activation of naïve T cells (details in methods section)
**Peptide-related impurity**	a molecular variant of the peptide drug resulting from synthetic peptide synthesis or storage (e.g., deletions, insertions, truncated, degraded and/or aggregate product)
**rDNA**	recombinant DNA
**RLD**	reference listed drug: this is the originator product for which a generic counterpart is made
**SCT**	salmon calcitonin.

## Data Availability

The original contributions presented in the study are included in the article/Supplementary Material; further inquiries can be directed to the corresponding author.
