# Supplementary material for: Assessing the immunogenicity risk of salmon calcitonin peptide impurities using in silico and in vitro methods
Source: Front Pharmacol. 2024 Aug 9;15:1363139. doi: 10.3389/fphar.2024.1363139 (PMC11341359; doi:10.3389/fphar.2024.1363139)
Supplement: Supplementary file 1 [file DataSheet1.PDF]

Supplemental Data for:

Assessing the Immunogenicity Risk of Salmon Calcitonin Peptide Impurities Using In Silico and In Vitro Methods

Brian J Roberts[1], Aimee Mattei[1], Kristina E. Howard[2], James L. Weaver[2], Hao Liu[3], Sandra Lelias[1] William D. Martin[1], Daniela Verthelyi[4], Eric Pang[3], Katie Edwards[5], Anne S. De Groot[1]

[1] EpiVax Inc, 188 Valley Street, Suite 424, Providence, RI 02909, USA

[2] Division of Applied Regulatory Sciences, Office of Clinical Pharmacology, Office of Translational Sciences, Center for Drug Evaluation and Research, U.S. Food and Drug Administration, 10903 New Hampshire Ave, Silver Spring, MD 20993, USA

[3] Division of Therapeutic Performance I, Office of Research and Standards, Office of Generic Drugs, Center for Drug Evaluation and Research, U.S. Food and Drug Administration, 10903 New Hampshire Ave, Silver Spring, MD 20993, USA

[4] Division of Biotechnology Review and Research III, Office of Biotechnology Products, Office of Pharmaceutical Quality, Center for Drug Evaluation and Research, U.S. Food and Drug Administration, 10903 New Hampshire Ave, Silver Spring, MD 20993, USA

[5] CUBRC,4455 Genesse Street, Suite 106 Buffalo, NY 12445, USA

Supplementary Table 1: Additional In Silico Tools Available for Commercial Use

T cell Epitope Prediction Tool	URL
EpiJen	http://www.ddg-pharmfac.net/epijen/EpiJen/EpiJen.htm
EpiMatrix	http://www.epivax.com/
IEDB	http://tools.iedb.org/main/tcell/
IMTECH	http://www.imtech.res.in/raghava/mhc
JanusMatrix	http://www.epivax.com/
MHC2Pred	http://www.imtech.res.in/raghava/mhc2pred/
MHCPred	http://www.ddg-pharmfac.net/mhcpred/
NeonMHC2	https://neonmhc2.org/
NetCTL	https://services.healthtech.dtu.dk/services/NetCTL-1.2/
NetMHC	https://services.healthtech.dtu.dk/services/NetMHC-4.0/
NetMHCIIpan	https://services.healthtech.dtu.dk/services/NetMHCIIpan-3.2/
PREDEP	http://margalit.huji.ac.il/Teppred/mhc-bind/index.html
RANKPEP	http://imed.med.ucm.es/Tools/rankpep.html
SVMHC	https://www.hsls.pitt.edu/obrc/index.php?page=URL1153429637
SVRMHC	http://svrmhc.biolead.org/
SYFPEITHI	http://www.syfpeithi.de/bin/MHCServer.dll/EpitopePrediction.htm

Supplementary Table 1: In silico tools utilized for analysis in this manuscript (EpiMatrix and JanusMatrix) were developed by EpiVax. Additional in silico tools that would allow researchers to obtain proximal results are provided.

Supplementary Table 2: 20 SCT Impurities Provided by the FDA OGD for In Silico Analysis

Input Name	Input Sequence	EMX Hits	EMX Score	JMX Score	Evaluated In Vitro
00_RLD_SCT	CSNLSTCVLGKLSQELHKLQTYPRTNTGSGTP	12	1.99	0.92	HLA and IVIP
01_CYSO7_SCT_7X	CSNLSTXVLGKLSQELHKLQTYPRTNTGSGTP	13	3.78	0.85	ND
02-LYS-AC11_SCT_11X	CSNLSTCVLGXLSQELHKLQTYPRTNTGSGTP	13	5.55	0.85	HLA Binding
03_Q14E_SCT	CSNLSTCVLGKLSEELHKLQTYPRTNTGSGTP	8	-5.01	0.13	ND
04-Q14PGLU_SCT_X14P	CSNLSTCVLGKLSPELHKLQTYPRTNTGSGTP	12	2.23	1.42	ND
05-HIS-O17_SCT_17X	CSNLSTCVLGKLSQELXKLQTYPRTNTGSGTP	13	2.55	0.54	ND
06-LYS-AC18_SCT_18X	CSNLSTCVLGKLSQELHXLQTYPRTNTGSGTP	13	3.33	0.46	IVIP
07_Q20E_SCT	CSNLSTCVLGKLSQELHKLETYPRTNTGSGTP	11	-0.92	0.73	IVIP
08-Q20PGLU_SCT_X20P	CSNLSTCVLGKLSQELHKLPTYPRTNTGSGTP	11	-1.28	0.73	ND
09_DES-THR21_SCT	CSNLSTCVLGKLSQELHKLQ-YPRTNTGSGTP	9	-4.06	1.44	HLA Binding
10_DES-THR25_SCT	CSNLSTCVLGKLSQELHKLQTYPR-NTGSGTP	13	4.96	0.85	ND
11_DES-ASN26_SCT	CSNLSTCVLGKLSQELHKLQTYPRT-TGSGTP	14	6.38	0.79	HLA Binding
12_ENDO-GLY28_SCT	CSNLSTCVLGKLSQELHKLQTYPRTNTGGSGTP	12	1.06	0.92	IVIP
13_DES-GLY28_SCT	CSNLSTCVLGKLSQELHKLQTYPRTNT-SGTP	12	2.92	0.92	ND
14_ENDO-SER29_SCT	CSNLSTCVLGKLSQELHKLQTYPRTNTGSSGTP	12	1.06	0.92	ND
15_DES29-32_SCT	CSNLSTCVLGKLSQELHKLQTYPRTNTG----	12	5.70	0.92	ND
16_DES-GLY30_SCT	CSNLSTCVLGKLSQELHKLQTYPRTNTGS-TP	13	4.70	0.92	ND
17_ENDO-THR31_SCT	CSNLSTCVLGKLSQELHKLQTYPRTNTGSGTTP	12	1.06	0.92	IVIP
18_DES31-32_SCT	CSNLSTCVLGKLSQELHKLQTYPRTNTGSG--	12	3.84	0.92	ND
19_DES31-32_SCT-OH	CSNLSTCVLGKLSQELHKLQTYPRTNTGSG--	12	3.84	0.92	ND
20_T31HYL_SCT_31X	CSNLSTCVLGKLSQELHKLQTYPRTNTGSGXP	12	1.99	0.92	ND

Supplementary Table 2. Overview of Input Sequences: Salmon Calcitonin Impurities. Amino acids in red represent a change from the 00_RLD_SCT sequence, - indicates a deleted amino acid residue. Where the amino acid is replaced with a red x, an unnatural amino acid or modification could not be directly modeled in silico and was replaced with the neutral place holder "X". An "X" neither promotes nor detracts from predicted binding in our algorithm. "ND" is for not done.

The purpose of this study was to demonstrate the use of orthogonal methods to assess the risk of synthetic peptide impurities. Therefore, of the 20 synthetic impurities evaluated in silico, three were selected for the class II HLA Binding Assay and three were selected for the IVIP naïve T cell assay. In a given 9-mer frame, amino acids in positions 1, 4, 6 and 9 face downwards towards the HLA binding groove and those in positions 2, 3, 5, 7 and 8 face upwards and interact with the T cell receptor. Impurities with modifications to HLA-binding residues were selected for binding assays, while impurities with modifications to TCR facing residues were selected for naïve T cell assays. The impurities were also selected for ease of synthesis. Impurities selected for in vitro assays were selected to represent a range of EpiMatrix scores representing immunogenicity risk potential.

PBMC were sourced from leukocyte reduction filters purchased from the Rhode Island Blood Center in Providence, RI. The Providence, RI population is evenly split between female (51.85%) and male (48.15%) residents. The median age in Providence is 29.8 years old, which is skewed towards younger residents (Supplemental **Figure 1A**). Comparing the donor cohort for the current study to the Providence RI population, males were represented at a slightly higher frequency than females (60% and 40% respectively). Overall, the average age of the donors was 53.4 years old, and the age range was 17-83 years old. The median age for the donor cohort was 53.5. Female donors were an average age of 49.1 years old with a range of 17-75 years old. The median female age was 50 years old. Male donors were an average age of 56.2 years old with a range of 42-83 years old. The median male age was 54 years old. Typically, the genders of the donor's populations are more balanced.

Supplementary Figure 1: PBMC Donor Cohort Population HLA DRB1 Types and Demographics

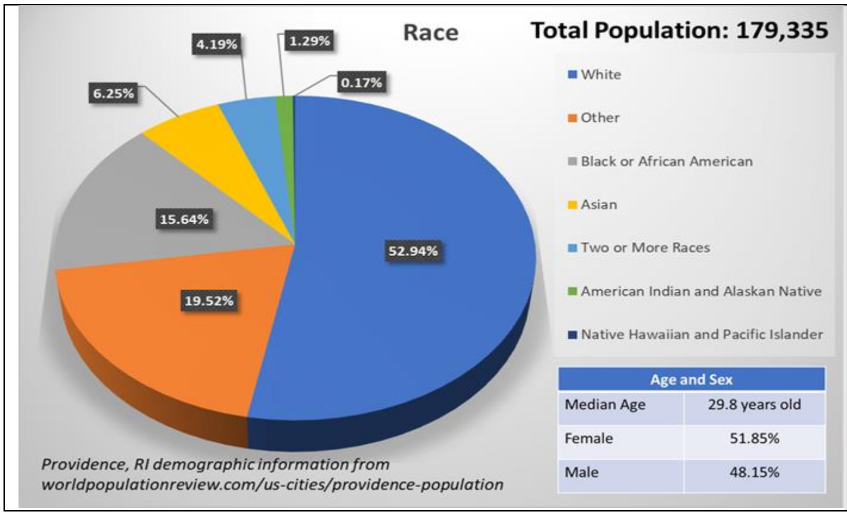

Supplemental Figure 1A: Population Demographics from Providence, RI. Citizens of Providence, RI are predominantly white, Black, African American, or other, with an even distribution of the sexes. Asians represent only 6.25% of the Providence, RI population explaining why DRB1*0901 are not represented by the donor cohort.

Donor ID	Age	Sex	HLA DRB1	
1	60	Male	03:01	03:01
2	41	Female	03:01	07:01
3	54	Male	14:01	15:01
4	54	Male	11:01	15:01
5	71	Male	07:01	11:01
6	51	Male	07:01	07:01
7	50	Male	04:02	13:05
8	56	Male	03:01	15:01
9	47	Female	07:01	11:04
10	47	Female	03:01	15:01
11	83	Male	15:01	15:01
12	42	Male	04:01	04:01
13	54	Male	04:05	14:01
14	53	Female	03:01	13:01
15	17	Female	07:01	07:01
16	54	Female	04:04	15:01
17	59	Female	04:02	10:01
18	51	Male	07:01	16:01
19	48	Male	11:01	15:01
20	75	Female	15:01	15:02

Donor Demographics			
	Female	Male	All
Percent	40%	60%	100%
Average Age	49.1	56.2	53.4
Median Age	50	54	53.5
Age Range	17-75	42-83	17-83

HLADRB1 Population Coverage	
Population	Coverage
East Asia	62.2%
Europe/North America	93.9%
Middle East	86.1%
North Africa	93.8%
South America	59.9%
Sub Saharan Africa	89.7%
Average	80.9
Standard Deviation	14.3

Supplemental Figure 1B: Donor Demographics. Males were slightly overrepresented in the cohort of donors. The average age of the donors was 53.4. A broad range of HLA DR alleles was represented in this cohort covering 80.9% of the alleles present in the global population.

Supplemental **Figure 1B** summarizes the HLA types of the donors evaluated in this study. HLA DRB1*15, the most represented supertype, expressed by 30.43% of the donors, is predominantly expressed by European, North American, and Sub-Sahara African populations. DRB1*07 is the next most represented supertype allele, expressed by 26.09% of the donors. DRB1*07 is predominantly expressed by Europeans, North Americans, and North Africans. DRB1*03 and DRB1*04 are the next most represented supertypes, each expressed by 21.74% of the donors. DRB1*03 and *04, are expressed primarily by the European and North American populations. DRB1*11, common in Sub-Sahara African and Middle Eastern populations, is expressed by 17.39% of the donors. DRB1*13, largely expressed by the Sub-Sahara African, North African, and Middle Eastern populations is represented in 8.70% of the donors.

DRB1*01, *08 and *09 are noticeably absent from the current donor cohort. DRB1*08 and *09 are primarily expressed by Central American and Asian populations. Donors expressing these alleles are typically quite rare in the Rhode Island population. Based on the Providence, RI population demographics, the Asian population represents only 6.25%

of the Providence population and therefore we expect these donors to be in the minority. DRB1*01, however, is most common in European, North American, and Middle Eastern populations and we would therefore expect this supertype to be more prevalent. Indeed, this supertype is commonly represented in previous donor cohorts from this region. Nearly 18% of the donors' express alleles were not covered by the nine supertype alleles, which include DRB1*10, *14 and *16. In summary, the panel of donors evaluated in this study are *primarily* represented by European, North American, African, and Middle Eastern populations, which is consistent with the population demographics for Providence, RI (Supplemental **Figure 1A**).

Supplementary Table 3: Response by PBMC Donor Individual Peptides

				API	Impurity			
Donor	Age<br>Sex	DRB1<br>Allele	EpiMatrix Hits Per Allele	Salmon Calcitonin	LYS-AC18_SCT	Q20E_SCT	ENDO-GLY28_SCT	ENDO-THR31_SCT
1	71	07:01	1	-	+	+	-	+
	Male	11:01	2					
2	51	07:01	1	-	-	-	-	-
	Male	07:01	1					
3	50	04:02	1	+	-	-	-	-
	Male	13:05	2					
4	56	03:01	2	+	+	+	+	+
	Male	15:01	1					
5	47	07:01	1	+	+	+	+	+
	Female	11:04	2					
6	47	03:01	3	+	+	+	-	+
	Female	15:01	1					
7	83	15:01	1	-	+	+	+	+
	Male	15:01	1					
8	42	04:01	1	-	-	-	+	+
	Male	04:01	1					
9	54	04:05	1	-	+	+	+	+
	Male	14:01	n/a					
10	53	03:01	3	-	-	+	+	+
	Female	13:01	2					
11	17	07:01	1	-	-	-	+	-
	Female	07:01	1					
12	54	04:04	1	-	-	-	-	-
	Female	15:01	1					
13	59	04:02	1	+	-	-	+	+
	Female	10:01	n/a					
14	51	07:01	1	-	+	-	-	-
	Male	16:01	n/a					
15	48	11:01	2	+	+	+	+	+
	Male	15:01	1					
16	75	15:01	1	+	+	+	+	+
	Female	15:02	1					
Total # of Positive Responses				7 / 16	9 / 16	9/16	10/16	11/16

Supplementary Table 4: Response by PBMC Donor RLD Spiked with Impurities

					Reference	Miacalcin® Spiked with Impurity			
Donor ID	Age / Sex	Donor	DRB1 Allele	EpiMatrix Hits Per Allele for SCT	Miacalcin® Injectable SCT	6 LYS-AC18_SCT	7 Q20E_SCT	12 ENDO-GLY28_SCT	17 ENDO-THR31_SCT
EV0275	60	4	03:01	2	-	-	-	-	+
	Male		03:01	2					137
EV0276	41	5	03:01	2	-	-	-	-	-
	Female		07:01	1					
EV0277	54	6	14:01	n/a	-	-	-	-	-
	Male		15:01	1					
EV0278	54	7	11:01	2	-	-	-	-	-
	Male		15:01	1					
EV0279	71	8	07:01	1	-	-	-	-	-
	Male		11:01	2					
EV0320	51	9	07:01	1	-	-	-	-	-
	Male		07:01	1					
EV0321	50	10	04:02	1	-	-	-	-	-
	Male		13:05	2					
EV0322	56	11	03:01	2	-	-	-	-	-
	Male		15:01	1					
EV0323	47	12	07:01	1	-	-	+	-	-
	Female		11:04	2			323		
EV0324	47	13	03:01	3	+	-	+	+	+
	Female		15:01	1	337		600	813	310
EV0325	83	14	15:01	1	-	-	+	-	-
	Male		15:01	1			1000		
EV0326	42	15	04:01	1	-	-	-	-	-
	Male		04:01	1					
EV0327	54	16	04:05	1	-	-	-	-	-
	Male		14:01	n/a					
EV0328	53	17	03:01	3	-	-	-	-	-
	Female		13:01	2					
EV0331	17	18	07:01	1	-	-	-	-	-
	Female		07:01	1					
EV0334	54	19	04:04	1	-	-	-	-	-
	Female		15:01	1					
EV0335	59	20	04:02	1	-	+	-	-	-
	female		10:01	n/a		80			
EV0337	51	21	07:01	1	-	+	+	+	-
	Male		16:01	n/a		700	1670	443	
EV0340	48	22	11:01	2	-	-	-	+	+
	Male		15:01	1				117	83
EV0342	75	23	15:01	1	+	-	-	-	-
	Female		15:02	1	210				
		Total # of Positive Responses			2 / 20	2 / 20	4 / 20	3 / 20	3 / 20
		Percentage of Responders			10%	10%	20%	15%	15%

Supplementary Table 4. High Level Summary of Individual Donor Responses to Miacalcin® spiked with impurities (positives highlighted in red). Numbers below “+” represented adjusted spot forming cells (SFC) per million cells.

Supplementary Figure 2: PBMC donor responses to SCT and impurities normalized to SCT

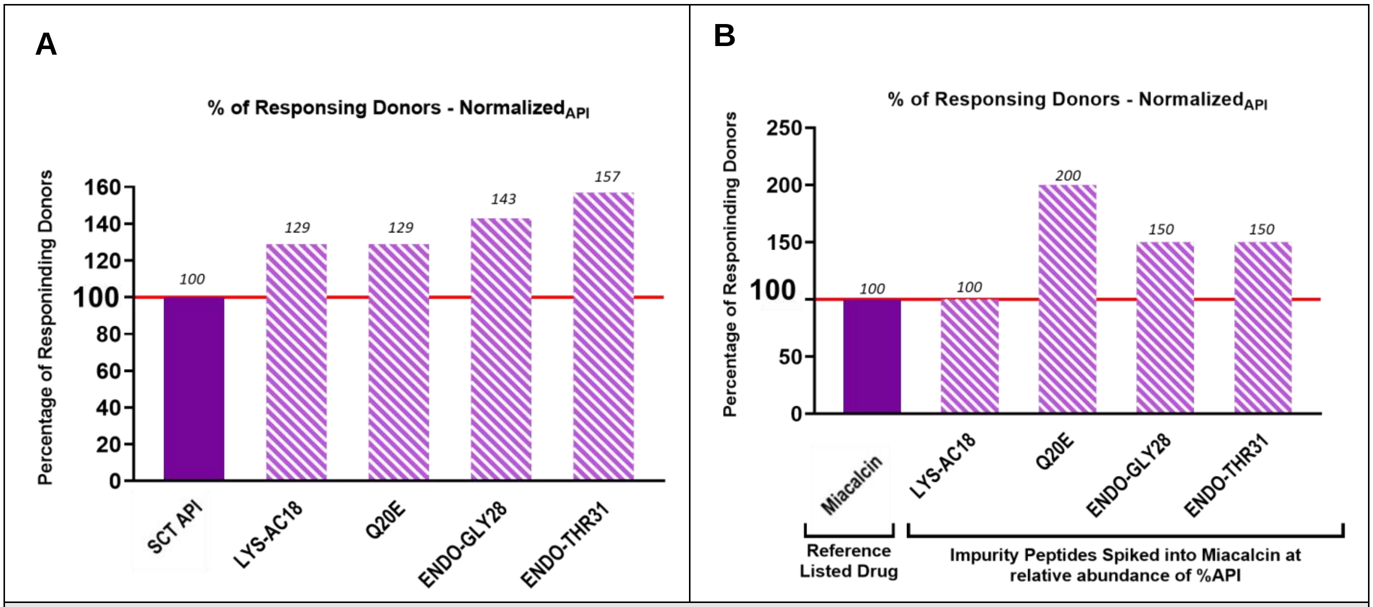

Supplemental Figure 2. PBMC donor responses to SCT and impurities normalized to SCT (A) Percentage of donor PBMC responding to the salmon calcitonin (API) versus each impurity. Data was normalized to the salmon calcitonin API (100%) to highlight the increase in the number of donor PBMC responding to each impurity compared to the API. Donor PBMC positivity include three criteria: (1) a INFg spot forming cells (SFC) >50, (2) Stimulation Index (SI) >2 and (3) a statistical difference between media and peptide stimulation as determined by student T test ($p < 0.05$). All peptides were evaluated at 20µg/ml, and the total number of donors tested was 16. Each of the four impurities elicited a higher percent response of PBMC donors when compared to the API peptide. **(B)** Percentage of donor PBMC responding to the Miacalcin® versus impurity-spiked Miacalcin samples. Data was normalized to the Miacalcin (100%) to highlight the increase in percent donor PBMC responding to the spiked samples as compared to the RLD alone. Donor PBMC positivity include three criteria: (1) a INFg spot forming cells (SFC) >50, (2) Stimulation Index (SI) >2 and (3) a statistical difference between media and peptide stimulation as determined by student T test ($p < 0.05$). The total number of donors tested was 20. Miacalcin® drug was evaluated at 20µg/ml. Impurity LYS-AC18_SCT was added at 0.24%, Q20E_SCT at 0.36%, ENDO-GLY28 at 2.62% and ENDO-THR31_SCT at 3.30% of SCT in Miacalcin. Three of the spiked impurities elicited a higher percent response of PBMC donors when compared to the Miacalcin alone.

Supplementary Discussion: Impact of formulation on cell health and functionality in in vitro T cell assays.

Initially the in vitro experiments were designed to confirm the capacity of the salmon calcitonin RLD, Miacalcin®, to induce a T cell response ex vivo and to (1) compare the response of the RLD to the RLD spiked with individual impurities, and (2) compare the SCT API peptide to individual impurity peptides for immunogenic risk potential. It was observed that the results were inconsistent with both the known immunogenicity of the drug product and the presence of a promiscuous immunogenic epitope revealed during

the in silico analysis. In these assays, only 10% of donor PBMC responded to the drug product as compared to 44% of the same cohort of donor PBMC that responded to the SCT API peptide. SCT in the formulated drug product and the API peptide were both evaluated at a concentration of 20.0 µg/ml (5.83µM) in vitro. One explanation may be that because the concentration of the API, and the donor PBMC cohort were the same for the two assays, it was reasoned that the excipients within the formulated drug product were impacting the health of the cells in vitro.

Specifically, the Miacalcin® product formulation buffer contains acetic acid, phenol, and sodium chloride. It was hypothesized that acetic acid, included as a protease inhibitor in Miacalcin®, impacted both cell health and function at the concentrations present in the formulation in vitro. Studies by Kitanovic et al (Kitanovic et al., 2012) support this assertion. They reported that intracellular accumulation of acetic acid creates a state of energy deficiency and nutrient starvation in cultures of *S. cerevisiae*. They further show that acetic acid directly influences the activity of several important metabolic enzymes including glyceraldehyde-3-phosphate dehydrogenase (GAPDH), pyruvate kinase and glucose-6-phosphate dehydrogenase (G6PDH).

During an immune response, activated T cells have increased metabolic needs to support expansion and effector function. Indeed, if glucose uptake is limited, and glucose levels fall below what is necessary for survival, apoptotic proteins such as Bcl-2 become active leading to cell death (Maciver et al., 2008). It was thus hypothesized that cells treated with the injectable Miacalcin® have an accumulation of acetic acid that is sublethal, not resulting in apoptosis, but sufficient to deprive T cells of the required energy necessary for proper effector function. Alternatively, acetic acid could be impacting the antigen presenting cells thus further limiting T cell responses in vitro. This would explain the limited in vitro response to Miacalcin® compared to the synthetic SCT peptide. Further study is required to confirm this hypothesis; however, this observation illustrates the importance of understanding the impact of drug product formulation on cell viability when designing assays to evaluate the immunogenicity of formulated drug products. For this reason, it may be beneficial to perform immunogenicity risk assessments comparing an individual API peptide to individual impurity peptides to understand the relative immunogenic risk potential of an impurity as compared to the API. It is recommended that such comparisons be performed with the API and impurity peptides at equivalent concentrations to better understand the immunogenic risk potential posed by the impurities. Manufacturers may also wish to evaluate the impurities at a lower concentration, consistent with their identified levels, alongside the equivalent concentration to the API as the relative abundance of an impurity within a product may impact the associated immunogenic risk.

References Cited

Kitanovic, A., Bonowski, F., Heigwer, F., Ruoff, P., Kitanovic, I., Ungewiss, C., et al. (2012). Acetic acid treatment in S. cerevisiae creates significant energy deficiency and nutrient starvation that is dependent on the activity of the mitochondrial transcriptional complex Hap2-3-4-5. *Front. Oncol.* 2, 118. doi: 10.3389/fonc.2012.00118.

Maciver, N. J., Jacobs, S. R., Wieman, H. L., Wofford, J. A., Coloff, J. L., and Rathmell, J. C. (2008). Glucose metabolism in lymphocytes is a regulated process with significant effects on immune cell function and survival. *J. Leukoc. Biol.* 84, 949–957. doi: 10.1189/jlb.0108024.
